# Supplementary figures and images for: Key performance indicators of COVID-19 contact tracing in Belgium from September 2020 to December 2021
Source: PLoS One. 2023 Oct 20;18(10):e0292346. doi: 10.1371/journal.pone.0292346 (PMC10588862; doi:10.1371/journal.pone.0292346)

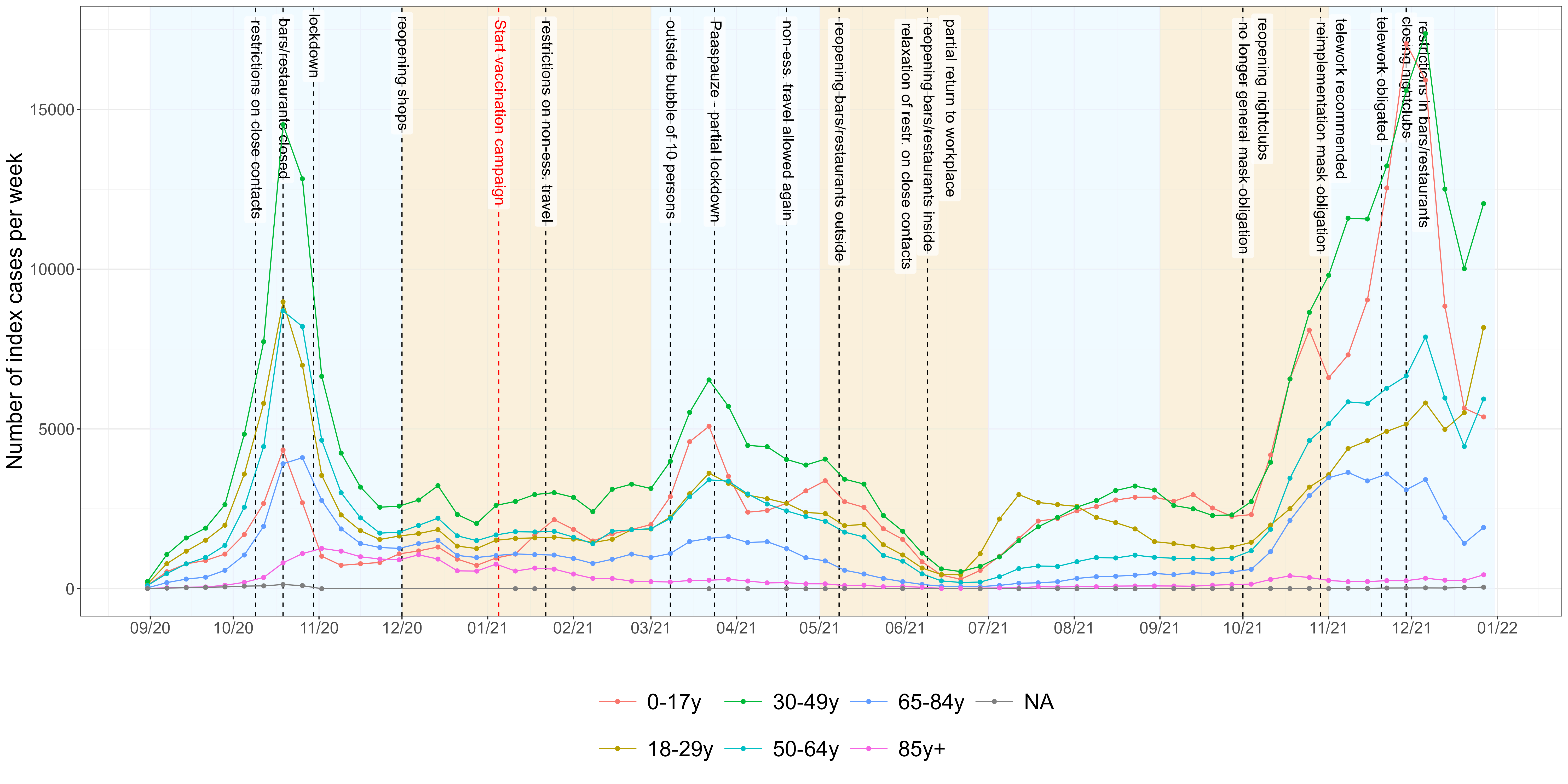

Supplement: S1 Fig — Number of index cases that were contacted during the period from September 2020 to December 2021 by age group, with an overview of the most influential control measures regarding social contacts. (TIF) [file pone.0292346.s001.tif]

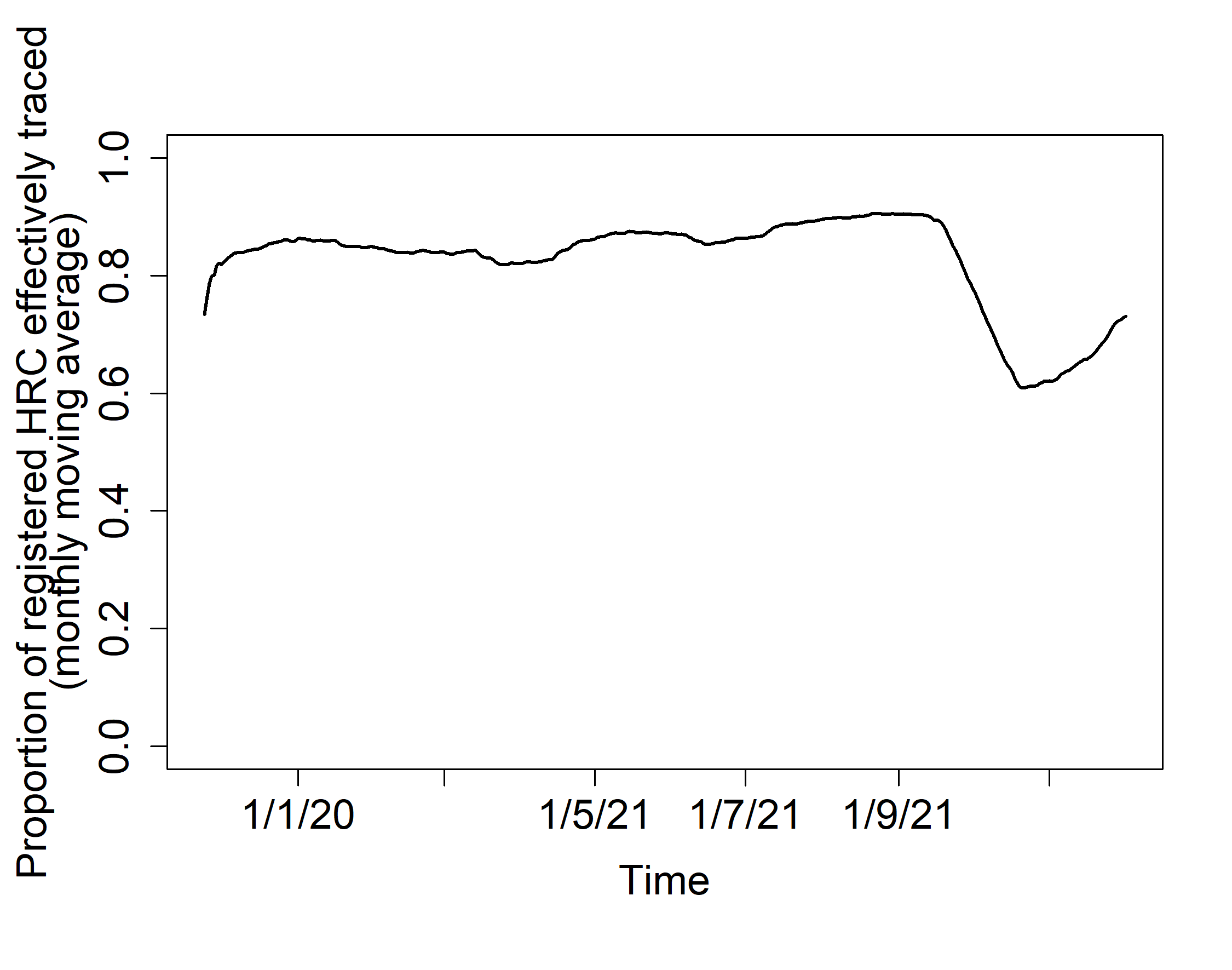

Supplement: S2 Fig — The proportion of registered high-risk contacts (HRC) that was effectively traced, over time (monthly moving average). (TIF) [file pone.0292346.s002.tif]

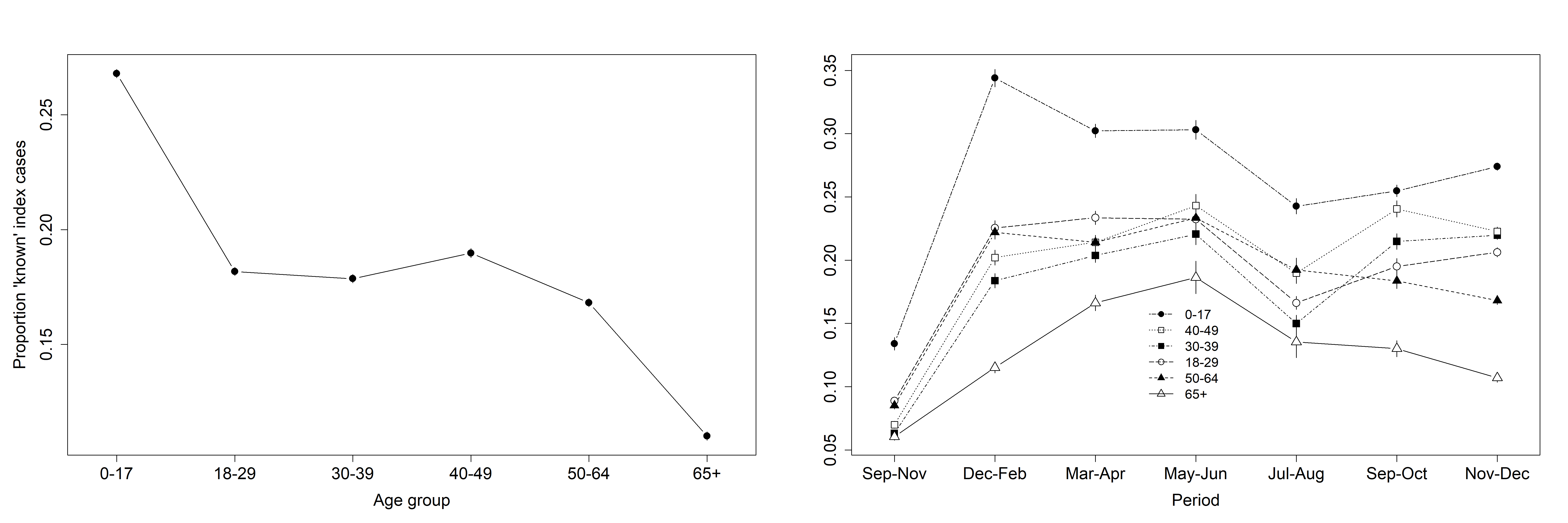

Supplement: S3 Fig — Evolution in the proportion of ‘known’ index cases by age group. Vertical bars represent the 95% confidence interval. (TIF) [file pone.0292346.s003.tif]

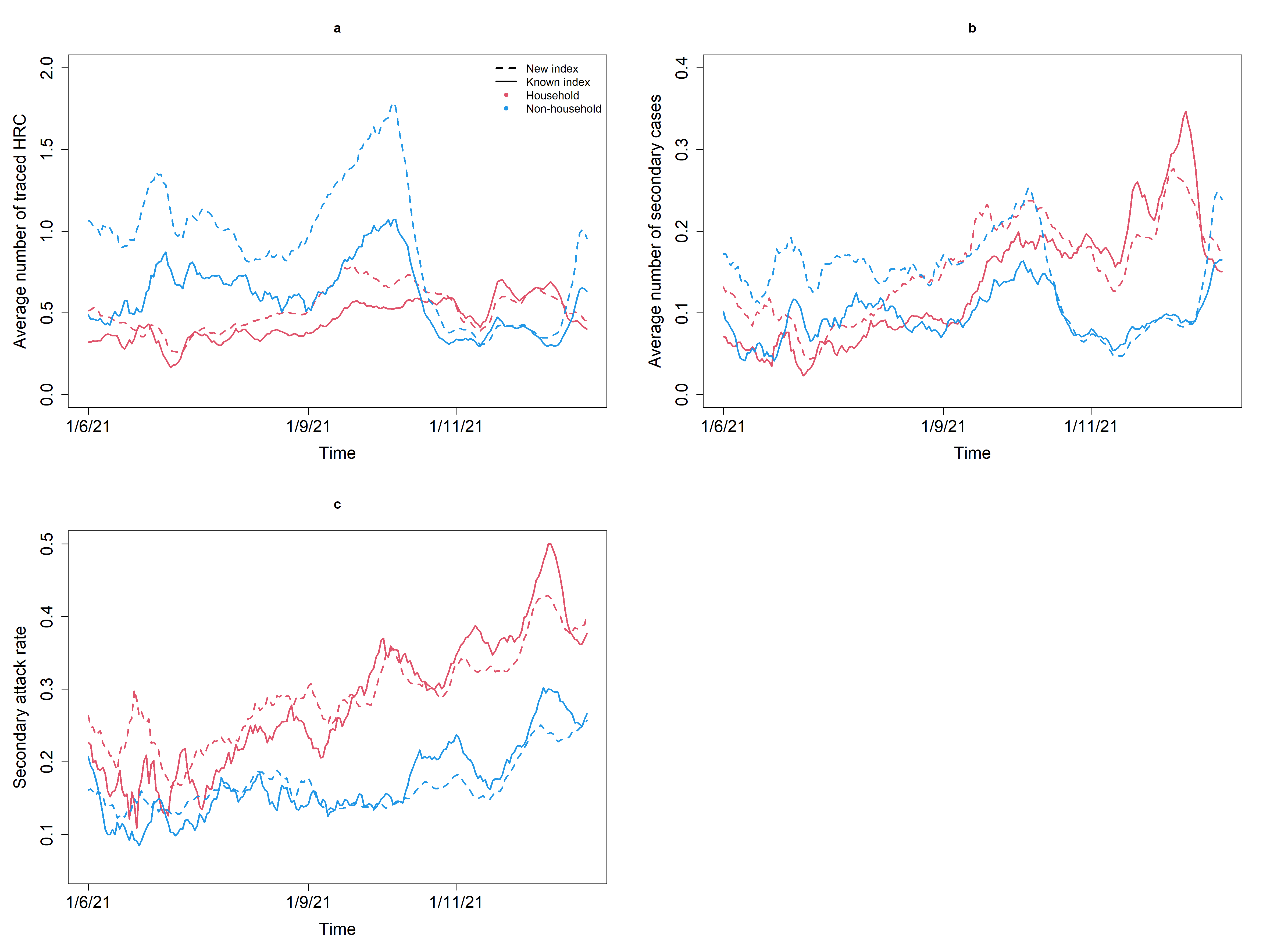

Supplement: S4 Fig — Evolution by household status in the (a) average number of traced HRC for ‘new’ and ‘known’ index cases, (b) average number of secondary cases for ‘new’ and ‘known’ index cases, and (c) secondary attack rate (SAR) among traced HRC of ‘new’ and ‘known’ index cases. (TIF) [file pone.0292346.s004.tif]

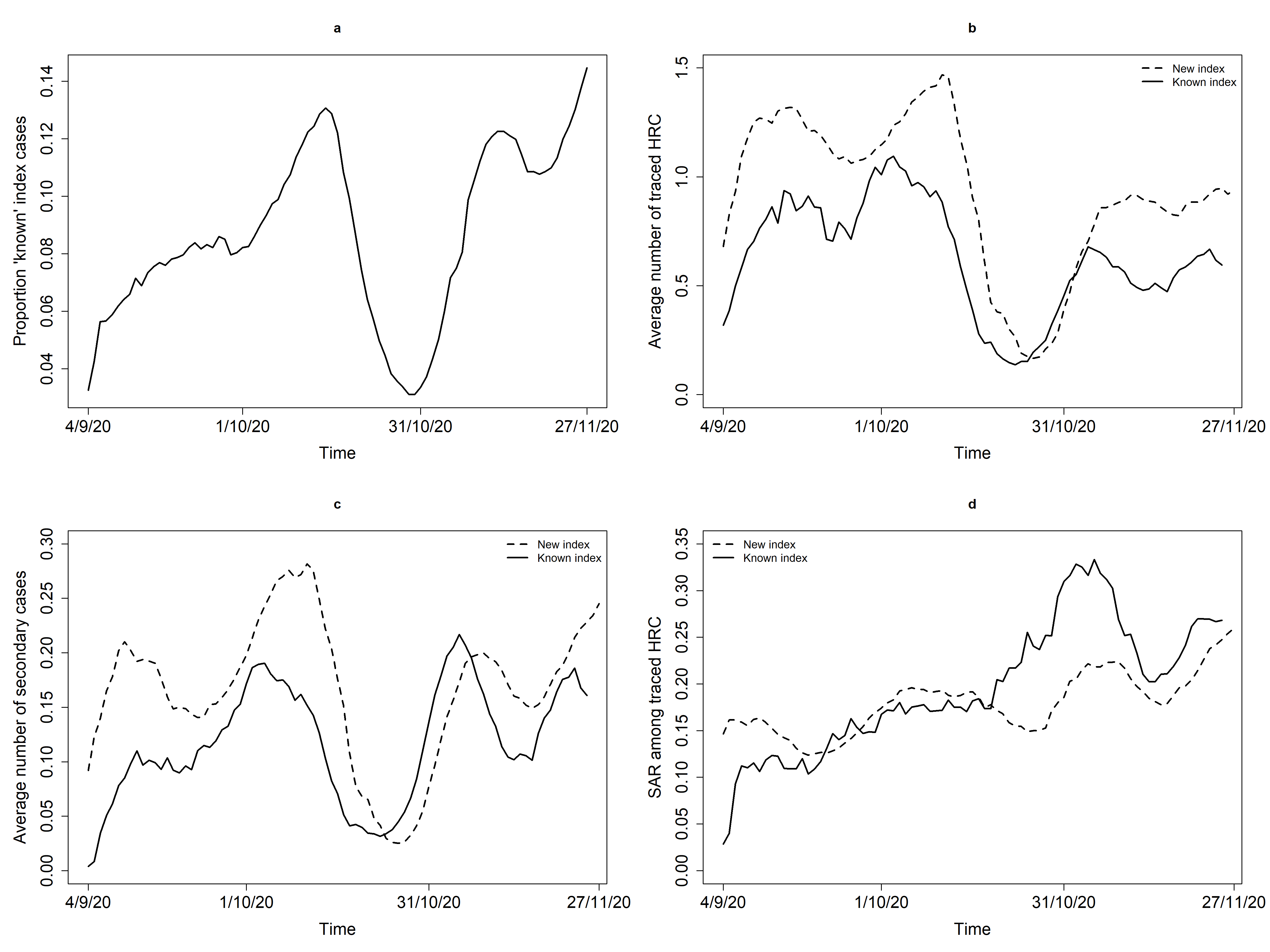

Supplement: S5 Fig — Evolution in the (a) proportion of index cases that were previously identified as a risk contact, (b) average number of traced high-risk contacts (HRC) for ‘new’ and ‘known’ index cases, (c) average number of secondary cases for ‘new’ and ‘known’ index cases, and (d) secondary attack rate (SAR) among traced HRC of ‘new’ and ‘known’ index cases, for the period from September to November 2020. (TIF) [file pone.0292346.s005.tif]
