# Supplementary material for: Key performance indicators of COVID-19 contact tracing in Belgium from September 2020 to December 2021
Source: PLoS One. 2023 Oct 20;18(10):e0292346. doi: 10.1371/journal.pone.0292346 (PMC10588862; doi:10.1371/journal.pone.0292346)
Supplement: S1 Table — Proportion of all index cases by province and age group. (PDF) [file pone.0292346.s006.pdf]

**S1 Table. Index case characteristics.** Proportion of all index cases by province and age group.

| | **0-17y** | **18-29y** | **30-39y** | **40-49y** | **50-64y** | **+65y** | **Unknown** |
|---|---|---|---|---|---|---|---|
| **Antwerp** | 0.052 | 0.040 | 0.038 | 0.034 | 0.038 | 0.023 | 0.000 |
| **Brussels Capital Region** | 0.053 | 0.047 | 0.045 | 0.040 | 0.037 | 0.020 | 0.000 |
| **East Flanders** | 0.042 | 0.032 | 0.031 | 0.030 | 0.033 | 0.024 | 0.000 |
| **Flemish Brabant** | 0.013 | 0.009 | 0.008 | 0.008 | 0.008 | 0.005 | 0.000 |
| **Limburg** | 0.022 | 0.016 | 0.016 | 0.016 | 0.019 | 0.012 | 0.000 |
| **West Flanders** | 0.032 | 0.024 | 0.023 | 0.022 | 0.028 | 0.022 | 0.000 |
| **Unknown** | 0.004 | 0.009 | 0.008 | 0.006 | 0.006 | 0.003 | 0.001 |
