# Supplementary material for: Key performance indicators of COVID-19 contact tracing in Belgium from September 2020 to December 2021
Source: PLoS One. 2023 Oct 20;18(10):e0292346. doi: 10.1371/journal.pone.0292346 (PMC10588862; doi:10.1371/journal.pone.0292346)
Supplement: Attachment — Submitted filename: Response to Reviewers.docx [file pone.0292346.s007.docx]

**Response to Reviewers**

Journal Requirements:

When submitting your revision, we need you to address these additional requirements.

1. Please ensure that your manuscript meets PLOS ONE's style requirements, including those for file naming. The PLOS ONE style templates can be found at

<https://journals.plos.org/plosone/s/file?id=wjVg/PLOSOne_formatting_sample_main_body.pdf> and

<https://journals.plos.org/plosone/s/file?id=ba62/PLOSOne_formatting_sample_title_authors_affiliations.pdf>.

**We have made sure that the revised manuscript adheres to the journal’s style requirements by using the provided LaTeX template.**

2. Please provide additional details regarding participant consent. In the ethics statement in the Methods and online submission information, please ensure that you have specified (1) whether consent was informed and (2) what type you obtained (for instance, written or verbal, and if verbal, how it was documented and witnessed). If your study included minors, state whether you obtained consent from parents or guardians. If the need for consent was waived by the ethics committee, please include this information.

If you are reporting a retrospective study of medical records or archived samples, please ensure that you have discussed whether all data were fully anonymized before you accessed them and/or whether the IRB or ethics committee waived the requirement for informed consent. If patients provided informed written consent to have data from their medical records used in research, please include this information.

**COVID-19 is a notifiable infectious disease, hence the data used in this study are part of mandatory reporting and they were pseudonymized before being made available. By law, these data can be used for statistical analyses (see** [**https://codex.vlaanderen.be/Portals/Codex/documenten/1018357.html - Artikel 7**](https://codex.vlaanderen.be/Portals/Codex/documenten/1018357.html%20-%20Artikel%207)**). We have mentioned on lines 156-157 in the ethics statement that “All personal data were encrypted by the Flemish Government's Department of Care before being made available for analysis”.**

3. Thank you for stating the following in the Competing Interests section:

“Naima Hammami is an employee of the Flemish Agency for Care and Health and Jorden Boone has been working as a consultant for the agency during the COVID-19 pandemic.
Niel Hens declares that the Universities of Antwerp and Hasselt have received funding for advisory boards and research projects of MSD, GSK, JnJ, Pfizer outside the proposed work. Niel Hens has not received any personal remuneration related to this work.
The other authors declare that they have no competing interests.”

We note that one or more of the authors are employed by a commercial company: Flemish Agency for Care and Health

a.            Please provide an amended Funding Statement declaring this commercial affiliation, as well as a statement regarding the Role of Funders in your study. If the funding organization did not play a role in the study design, data collection and analysis, decision to publish, or preparation of the manuscript and only provided financial support in the form of authors' salaries and/or research materials, please review your statements relating to the author contributions, and ensure you have specifically and accurately indicated the role(s) that these authors had in your study. You can update author roles in the Author Contributions section of the online submission form.

Please also include the following statement within your amended Funding Statement.

“The funder provided support in the form of salaries for authors [insert relevant initials], but did not have any additional role in the study design, data collection and analysis, decision to publish, or preparation of the manuscript. The specific roles of these authors are articulated in the ‘author contributions’ section.”

If your commercial affiliation did play a role in your study, please state and explain this role within your updated Funding Statement.

b. Please also provide an updated Competing Interests Statement declaring this commercial affiliation along with any other relevant declarations relating to employment, consultancy, patents, products in development, or marketed products, etc.

Within your Competing Interests Statement, please confirm that this commercial affiliation does not alter your adherence to all PLOS ONE policies on sharing data and materials by including the following statement: "This does not alter our adherence to  PLOS ONE policies on sharing data and materials.” (as detailed online in our guide for authors <http://journals.plos.org/plosone/s/competing-interests>) . If this adherence statement is not accurate and  there are restrictions on sharing of data and/or materials, please state these. Please note that we cannot proceed with consideration of your article until this information has been declared.

Please include both an updated Funding Statement and Competing Interests Statement in your cover letter. We will change the online submission form on your behalf

**We want to point out that the Flemish Agency for Care and Health (which changed its name since July 1^st^ to Department of Care, we have adapted this in the manuscript accordingly) is not a commercial company but a governmental agency at regional level.**

Reviewer #1: Overall, the authors address a relevant topic in the field of epidemiological surveillance and intervention in public health. The key performance indicators are well suited, and the division between cases that were previously identified as close contacts and those that were not yet known is adequate. Although other papers have already suggested the effectiveness of contact tracing, there is an abundance of modelling studies and a lack of community and real-world studies. The former are dependent on a plethora of premises that often fail to represent reality, whereas the latter reflect reality with all its complexities, from which I believe this manuscript could be relevant.

**We thank the reviewer for their interest in our study and for their thorough review.**

The manuscript, however, is poorly designed. The introduction is far too large, and could be reduced to half the size without losing any of its relevant points. In fact, its length – a problem applicable to the entire paper – draws the reader off the idea the authors are trying to communicate.

**Following suggestions of the reviewer we have rewritten the Introduction and substantially reduced its length.**

The title and the abstract focus mainly on the effectiveness of contact tracing. That should be the main aim of the paper, as given by the objectives in the end of the introduction. However, when one reaches the Results and Discussion section, it is mingled with secondary analysis (age and close contacts) which are more dubious, and definitely less relevant. The authors focus too much on the statistical presentation of secondary analysis, without much consideration for the message they are trying to pass and the epidemiological implications of their choices. Also, the authors rely on their figures heavily and provide few data on the results text section, which is a major limitation. The inclusion of 6 figures, with no tables (such as baseline characteristics) suggests that authors were unable to focus their message on the essential. In fact, after reading the manuscript several times, it seems that authors changed their focus midway, which denotes a frail conception.

**We hope that by removing the secondary analysis and by including tables displaying numeric results, as suggested by the reviewer, the aim of our manuscript which was indeed to focus on the effectiveness of contact tracing is now more clear. Additionally, we have made substantial edits to the manuscript text, aiming to give more attention to the effectiveness as indicated by the KPIs. We have also added a paragraph to the Discussion on lines 330-334 where we acknowledge that an individual-based model could be used to further quantify the impact in terms of what would have happened if no contact tracing would have been in place. Such a model exists for Belgium, but would need non-trivial extensions in order to model contact tracing scenarios as they were implemented in real-life. This approach was beyond the scope of the present study but would be an interesting direction for future research.**

Notes by parts

Abstract

“(…) fewer close contacts as well as fewer secondary cases and a lower secondary attack rate (…)”: the absence of quantitative data is a major limitation. Even in the abstract, major results ought to be given.

**We thank the reviewer for pointing this out and we have now added quantitative results in the Abstract.**

The conclusion that “…individuals aware of their exposure to SARS-CoV-2 seemed more reserved in their social contact behavior” is, at best, an euphemism for the fact that they were placed under quarantine and had less contacts to report, which is the point of contact tracing and quarantine. This is no direct consequence of the results here provided and should not be included.

**We agree with the reviewer that individuals aware of their exposure and reached by the contact tracing were put in quarantine and hence reported less contacts. We have left out this part of the sentence in the abstract. However, we would like to point out that the significantly lower secondary attack rates among ‘known’ cases from December 2020 until August 2021 may indicate that these individuals were more careful with the contacts they did have, and this is still mentioned in the Discussion on lines 233-236.**

Introduction:

Lines 8-9: “When restrictions were relieved, countries implemented contact tracing…”. As I understand from the manuscript, this was how Belgium tackled the pandemic; however, in many settings that was not so. In my country, contact tracing was operational prior to the pandemic (for other infectious diseases) and was implemented prior to, during, and after all lockdowns.

**We thank the reviewer for bringing this to our attention and have adapted this sentence on lines 7-8 as follows: “In the philosophy of having person-tailored quarantine measures, COVID-19 contact tracing was implemented in several countries.”**

Lines 12-14: conceptually, other problems like asymptomatic cases transmitting disease, short incubation periods, acute disease, are all characteristics affecting the effectiveness. Also, a reference is missing.

**We have now briefly mentioned this in the Discussion on lines 288-289 as follows: “In addition to operational challenges and disease characteristics, such as asymptomatic infections, …”. On line 13 we also added a reference to Juneau et al. (2023) who performed a systematic review regarding the effectiveness of COVID-19 contact tracing.**

Lines 20-26: Superfluous. I understand the relevance of the cited papers, yet the previous sentences already established that potential benefits of contact tracing. The authors may opt to include reference 9 in the end of line 20.

**We have replaced this part by the single sentence: “However, there is a need for empirical studies on the effectiveness of contact tracing” on lines 16-17.**

Lines 27-34: Same thing. Interesting results to support the effectiveness of contact tracing, but which add nothing to what was previously established. Also, in the first line “It is not trivial…” it seems to this reviewer that there would be no need to compare regions with and without contact tracing, because you are comparing cases subjected to contact tracing to “new” cases. That distinction is already made. Therefore, this section is redundant.

**We apologize for the confusion. This statement is more about quantifying the effectiveness of contact tracing in terms of the number of cases averted, which is not trivial without a reference region or period and cannot be done based on the distinction between ‘new’ and ‘known’ cases. We have adapted this paragraph on lines 18-21 as follows: “In the absence of a reference period or region, it is not trivial to quantify the effectiveness of contact tracing in terms of the number of cases and deaths averted. However, the potential impact of contact tracing on onward transmission can be evaluated using several key performance indicators (KPIs).”**

Lines 42-onwards: These are methods. It includes setting, time window and context. I would move this to the methods sections (albeit in fewer sentences) and conclude only with the objectives

**We have adapted this sentence on lines 33-40 as follows: “In this study, we used contact tracing data from Belgium (Flemish and Brussels-Capital region) collected between September 2020 and December 2021 to investigate (1) the proportion …”. The other information regarding study setting etc. was moved to the Methods section.**

Lines 55-56: “We investigated several key indicators and discuss their opportunities and pitfalls”. Meaningless sentence. It is relevant to know which key indicators were selected, which the authors provide in the next sentence.

**We have adapted this sentence as mentioned in our reply to the comment above.**

Lines 59-60: attack rate?

**We have adapted this sentence on lines 38-39 as follows: “…, (4) the ratio of potential infections over contacts, i.e. secondary attack rate, ...”**

Methods:

Lines 75-80: There is no specification as to what type of measures were implemented for high and low-risk contacts. One assumes that high-risk patients were placed under quarantined, whereas low-risk were either subjected to active surveillance, or were disregarded due to lack of resources. The end sentence (between brackets) is redundant and should be deleted.

**Different testing and quarantine measures were implemented for high- and low-risk contacts, and these measures varied during the study period. For example, on 25 January 2021 it was decided that HRC had to get tested twice instead of only once (https://covid-19.sciensano.be/nl/procedures/historiek-wijzigingen). We have removed the sentence between brackets and added the following on lines 55-57: “Testing and quarantine measures for HRC varied throughout the study period, and LRC were instructed to heighten their vigilance and undergo testing if they exhibited any symptoms.”**

Lines 81-87: Although the authors take a lot of consideration to distinguish between reported, registered and traced contacts. I understand that registered contacts were not necessarily traced, which happened frequently for many reasons. However, it is not clear to this reviewer the difference between reported and registered. Was it possible for a case to report 10 contacts, but the interviewer would only register 8 in the system?

**Reported contacts were registered following specific guidelines throughout the study period. Reasons for not registering reported contacts include being reported by more than one index case (hence already registered), being a household member that is already quarantined, or no recollection of the contact’s name. These have been mentioned in the manuscript on lines 65-71.**

Lines 99-100. Other (referenced) papers opted to exclude these cases and contacts.

**We have clarified that we did not include contacts occurring within a collectivity on lines 78-80 as follows: “Contacts occurring within a collectivity were followed up by the responsible medical services, these data were not available to us and hence not included in our study.”**

Line 126 and line 146: there appears to be a contradiction. The transmission line is said to be constructed for cases and their reported index case if the test was positive 21 days before or after the index case. In the end of the paragraph, it is claimed that the serial interval was constrained to -5 to 21 days. Is this referring to different steps of the process?

**We apologize for the confusion. The transmission line list is based on the *date of positive test*, and we included indexes and their contact as potential transmission pairs if their positive test dates were within 21 days of each other. In this way, we account for the uncertainty in directionality of transmission, for example a secondary case could have been tested before their true infector and hence would appear as an ‘index’ in our dataset. The serial interval on the other hand is based on *symptom onset dates*, which were not available for all individuals. This calculation therefore includes only those pairs for which both individuals had reported their time of symptom onset, and we constrained the serial interval to lie in the biologically plausible interval of -5 to 21 days. It is however possible that the interval between test dates of the index and contact is <(-5) days, and using this as a lower limit for the construction of the transmission line list (and hence for the transmission matrices) may be too restrictive.**

**We have clarified this in the manuscript as follows on lines 117-120: “A transmission line list was conceived by linking infected contacts to their reported index case if positive tests occurred in a 21-day time span, assuming that transmission could have occurred in either direction.” and on lines 146-147: “Individuals for whom the timing of symptom onset was not available, because they did not report it or were asymptomatic, were excluded from this calculation.”**

**We’d also like to note here that if we would have constructed the transmission line list based on the date of symptom onset, we would have excluded half of the potential transmission pairs because one or both individuals had a missing date of symptom onset.**

Results:

The presentation of results is absurd, and needs a complete overhaul.

Lines 143-136: Most likely estimating the speed of contact tracing (which is relevant) rather than transmission.

**We agree that fluctuations in the serial interval over time can be partly attributed to changes in the speed of contact tracing, but this would not explain the difference between ‘known’ and ‘new’ cases. ‘Known’ cases were however quarantined earlier in their infectious period due to their high risk of exposure, which will contribute to a shorter serial interval. This is mentioned in the Discussion on lines 280-283.**

Line 168: To use percentages to provide numbers larger than 100% is a poor choice to present data. Additionally, I can’t figure where this number comes from. 416 645 unique cases represent 41.5% of all included cases. Even if I may understand there is a dot/comma missing in virtually every data provided, still the number is not correct.

**We apologize for an error in the formatting of decimal numbers throughout the manuscript and the confusion this has brought on. This has now been corrected. Out of the 987 210 unique individuals included in the data, 416 645 were linked to traced contacts, i.e. 42.2% of all available index cases.**

Lines 168-170: If the result you have is summarized in a figure, with no more than a sentence claiming the same as the legend, then it is superfluous. It is an interesting image to present in a workshop or seminar, eventually, but it adds nothing here.

**We agree with the reviewer that this figure should not have been included in the main text, but we do believe it could be relevant to readers who want to know more about specific control measures and incidence of cases during the study period. We have therefore moved this figure to the Supporting Information (S1 Fig), and will leave it at the Editor’s discretion whether or not to keep this.**

Line 178: It seems like a proportion, and hence could not be 191%. Assuming 19.1%, this is different from the result given in the abstract (20%).

**We apologize for the confusion, indeed this should have been 19.1%. We have also adapted the abstract to mention the exact proportion.**

Line 180-181: The proportion seems stable, except for the first point. Rephrase.

**We have adapted this sentence on lines 176-178 follows: “… fluctuated around 20% for the remaining study period, being highest (24.8%) during May-June 2021 and lowest (18.9%) during July-August 2021.”**

Lines: 188-189: Discussion!

**This sentence was moved to lines 236-238 in the Discussion.**

Figure 2: Where are the confidence intervals? Are these results significant?

**The vertical bars in Figure 2 (now Figure 1) represent the 95% confidence intervals. Due to the large numbers however, these bars are not always (clearly) visible. We have added Table 1 displaying these results numerically. There it can be seen that the average number of traced HRC and secondary cases differs significantly between ‘known’ and ‘new’ cases in each time period. The SAR among traced HRC is significantly higher for ‘new’ cases from December 2020 until August 2021.**

Line 199: 137%. Same issue.

**We apologize for the confusion and have corrected this to 13.7%.**

Line 200: 4 points below vs 3 points. Virtually similar. Dubious interpretation.

**Discarding the first point in Sep-Nov 2020 (for reasons explained in the manuscript), the SAR was significantly lower among ‘known’ index cases from Dec ’20 to Aug ’21. We hope the addition of numeric presentation of these results in Table 1 helps to clarify this.**

Line 207: S4 figure is absolutely redundant, even as a supplement.

**This figure has now been removed from the Supporting Information.**

Line 208-220: This paragraph is a mix of methods, results and discussion. Needs complete rewriting.

**This paragraph has been removed and its contents now placed within the appropriate sections.**

Figure 3: Redundant. Doesn’t seem to add to the discussion.

**We appreciate this comment but we do believe these delay distributions are relevant to obtain insight in the speed of contact tracing. For now we have kept this Figure and included some additional interpretation in the Results section and in the Discussion on lines 238-239, but we will leave it at the Editor’s discretion whether or not to keep this.**

Line 234-242: No clear reason for the stratification per month. Given the information which is provided in the discussion, would it be not more useful to stratify per variant? Also, the differential proportion of vaccination during the time period is never taken into account, statistically. Another option was to stratify per strategy, as given in the first paragraph of the discussion.

**Since sequencing information was not available for all index cases, stratification per variant was approximated by stratification per month during which each VoC was the dominant strain. We have clarified this in the Methods section on lines 134-136 as follows: “Since only limited sequencing information was available, we analyzed contact and transmission matrices for specific months during which each VoC was the dominant circulating strain.”**

**In Belgium, the vaccination campaign in the general population started early 2021, hence for the largest part of the time period considered here vaccination coverage was quite low. Unfortunately we do not have any data available regarding vaccination status of the index cases and their contacts. A previous study in which these data were available reported that by the end of September 2021, only 36% of index cases in the contact tracing database were fully vaccinated. We have included this in the Discussion section on lines 277-279 as follows: “A previous study that linked Belgian contact tracing data with vaccine uptake reported that only 36% of the index cases were fully vaccinated by the end of September 2021.”**

Lines 243-250: It is unclear to this reviewer the relevance of this analysis. Given the bias acknowledged by failing to include older people, the results are inconsequential. I would delete this sub-analysis.

**As recommended by the reviewer we have deleted this secondary analysis from the manuscript.**

Line 336: One third of HRC were excluded. This is tremendous, and should have 1) led the authors to focus more on the effectiveness of contact tracing and quarantine, which is relegated to a secondary position after the introduction, and 2) to provide some comparison between included and excluded HRC, to analyze whether the included are representative of the population and, if not, how not so.

**We hope that the revised manuscript more clearly emphasizes the effectiveness of contact tracing. Unfortunately, no data on characteristics such as age, gender, or region are available for the HRC with missing national registry numbers. It is therefore not possible to compare those to the included HRC.**

Discussion

Lines 266-267: Individuals attending the same gathering were classified as HRC <- highly relevant data that should be given in the methods section!

**We have now moved this part to the Methods section on lines 88-93.**

Lines 293-295: See previous comment.

**As mentioned in our reply to the previous comment, vaccination coverage in the general population during our study period was quite low, and even lower among index cases in the contact tracing database as revealed by a study in which data on vaccination status were available.**

Line 301-306: This suggests that analysis should have been stratified by variant, which is meaningful, rather than monthly, which is comparing completely different scenarios.

**As mentioned in our reply to the comment above, the influence of different VoCs was investigated based on the months during which each of these VoCs was dominant since we had only limited sequencing information available.**

Lines 307-317: It is not clear why this paragraph is included, at all.

**We have removed this paragraph and added the reference to this study elsewhere.**

Limitations should note that there may be a differential misclassification of ‘exposure’, as always happens in cases like this: all ‘known’ cases were definitively previously traced, whereas ‘new’ cases are likely to include a few cases who were quarantined, but whose quarantine was unknown to the researchers/public health authorities.

**We agree with the reviewer that this limitation should be mentioned, and this will be especially the case for household contacts, which were often not registered as HRC if they were already quarantined. We have added this on lines 309-312 as follows: “In addition, whereas all `known' cases have been previously traced and quarantined, `new' cases are likely to include some cases who were quarantined but not registered as HRC, especially if they were household contacts of an index case.”**
